# Implementation of the injury prevention exercise programme *Knee Control+*: a cross-sectional study after dissemination efforts within a football district

**DOI:** 10.1136/ip-2023-044863

**Published:** 2023-05-31

**Authors:** Hanna Lindblom, Sofi Sonesson, Josefin Forslind, Markus Waldén, Martin Hägglund

**Affiliations:** 1 Unit of Physiotherapy, Department of Health, Medicine and Caring Sciences, Linköping University, Linköping, Sweden; 2 Sport Without Injury ProgrammE (SWIPE), Department of Health, Medicine and Caring Sciences, Linköping University, Linköping, Sweden; 3 Capio Ortho Center Skåne, Malmö, Sweden

**Keywords:** training, uptake/adherence, implementation / translation, sports / leisure facility, program evaluation

## Abstract

**Background:**

The Reach, Effectiveness, Adoption, Implementation, and Maintenance (RE-AIM) framework can be used for evaluation of implementation initiatives in sports injury prevention. The aim was to evaluate the implementation of the injury prevention exercise programme *Knee Control+* among amateur clubs and coaches in one regional football district using all five dimensions of the RE-AIM framework.

**Methods:**

Dissemination of *Knee Control+* during the 2021 season with information and webinars within one regional football district. This was followed by a cross-sectional study with questionnaires to club personnel and coaches after the season.

**Results:**

The reach of *Knee Control+* was fair to high, 83% of club personnel and 66% of coaches knew about the programme. 41% of club personnel and 51% of coaches had adopted it. Perceived programme effectiveness was high (6 on a 1–7 Likert scale) among coaches. Regarding implementation and maintenance, 27% of club personnel had informed coaches about *Knee Control+* and 57% planned to inform coaches. The coaches had implemented the programme mainly as recommended, but half used the programme once per week or less. Intention to maintain use of the programme was high (7 on a 1–7 Likert scale) among coaches.

**Conclusion:**

The reach of *Knee Control+* was fair to high, and adoption was fair in clubs, but there was a lack of policies for preventive training. Active strategies probably need to accompany dissemination of programme material. Reach, perceived effectiveness, adoption, implementation and planned maintenance were positive among coaches, but further studies are needed to analyse long-term maintenance.

**WHAT IS ALREADY KNOWN ON THIS TOPIC**

- Injury prevention exercise programmes are efficacious in preventing sports injuries.
- Effectiveness of injury prevention exercise programmes outside the controlled setting of a randomised trial is lower compared with the efficacy shown in randomised controlled trials.
- High-quality implementation of injury prevention exercise programmes is challenging.

**WHAT THIS STUDY ADDS**

- Reach of *Knee Control+* was fair to high and adoption was fair among clubs and coaches after programme dissemination at the start of the season. Perceived effectiveness was high among coaches.
- Implementation, in terms of whether *Knee Control+* was used as intended, seemed positive regarding training duration and dosage, whereas training frequency needs improvement.
- High programme reach indicates that our dissemination strategies were sufficient to reach clubs. Low prevalence of formal policies for injury prevention and low use of *Knee Control+* programme material suggest that further strategies are necessary to support club injury prevention programme implementation.

**HOW THIS STUDY MIGHT AFFECT RESEARCH, PRACTICE OR POLICY**

- *Knee Control+* seemed easy to use in terms of the included exercises and set-up, but teams may need more support to achieve sufficient training frequency and fidelity with the programme components.
- Future studies should follow-up on the maintenance of *Knee Control+* use over time.
- Active strategies, such as offering workshops on-site and forming policies supporting injury prevention, may be necessary to improve implementation of injury prevention exercise programmes in clubs.

## Introduction

Injury prevention exercise programmes (IPEPs) are efficacious in preventing sports injuries in randomised controlled trials (RCTs),^1–5^ especially among the most adherent players.^6–9^ However, this injury risk reduction is compromised when studying programme effectiveness outside the controlled context of an RCT.^10 11^ For the IPEP *Knee Control* a 64% reduction in the rate of anterior cruciate ligament injuries was shown in an RCT,^1^ whereas a study on insurance data the years following nationwide implementation of *Knee Control* only showed a 13% reduction in cruciate ligament injuries.^10^ Coaches may not know about and use the programme or may modify programme content or dosage,^12–14^ thereby potentially limiting the effect. A further challenge is to maintain programme use over multiple seasons. *Knee Control+* was developed from the *Knee Control* programme^1 15^ and a preliminary version named extended *Knee Control*.^16^ The intention with *Knee Control+* was to improve programme feasibility, making programme exercises and set-up easier to use and adaptable to different age and playing levels, and avoid arbitrary modifications of programme content or dosage seen in *Knee Control*, and thereby facilitate programme use and maintenance. A recent randomised trial on the extended *Knee Control* confirmed programme efficacy with 29% lower hamstring, knee and ankle injury incidence (combined) and 17%–26% lower weekly prevalence in the same locations compared with teams that already used self-selected preventive exercises or teams that used a groin injury prevention programme.^16^ In addition to studying programme efficacy in randomised trials, research is needed on dissemination and implementation in routine practice^17^ to make sure that the best available scientific evidence is implemented.

In this study, ‘dissemination’ is used to describe the active spread of interventions, whereas ‘implementation’ is defined in line with Rabin and Brownson^18^ as ‘the process of putting to use or integrating evidence-based interventions within a setting’. The Reach, Effectiveness, Adoption, Implementation, and Maintenance (RE-AIM) framework^19 20^ may be used as a framework to evaluate implementation.^21^ Most sports injury prevention studies report IPEP effectiveness, whereas few studies report programme adoption and maintenance.^22 23^ Only a few studies have covered all five dimensions of the RE-AIM framework.^12 21^ *Knee Control+* was developed from the *Knee Control* programme specifically to facilitate adherence, but its implementation has not been evaluated outside the controlled context of a randomised trial. Better knowledge of programme implementation may benefit further research and dissemination strategies for *Knee Control+* and other similar IPEPs.

### Aim

The aim was to evaluate the implementation of the IPEP *Knee Control+* among amateur clubs and coaches in one regional football district using all five dimensions of the RE-AIM framework.

## Methodology

### Study design

This study used a cross-sectional design to evaluate the implementation of *Knee Control+* within one regional football district in Sweden via web-based questionnaires after the 2021 football season. The manuscript was checked against the Strengthening the Reporting of Observational Studies in Epidemiology guidelines.^24^

The 2021 football season in Sweden was affected by the COVID-19 pandemic and the accompanying authority restrictions and recommendations so the competitive season was postponed approximately 6 weeks and started end of May for the adolescent series and 1 June 2021 for adults in the amateur series.

### Study population

All football clubs within one regional district football association (FA), Östergötland, out of 24 regional districts in Sweden (with almost 3000 football clubs), and all teams with registered players ≥12 years of age in the same district were targeted with dissemination initiatives. In the senior leagues, coaches for male (3rd–8th leagues), female (3rd–6th leagues) and the male development series were eligible. All players were amateur players, that is, did not have a written contract and were not paid for more than his/her expenses. The same population received questionnaires at the end of the season. Contact information (emails and telephone numbers) for clubs and coaches was collected via the clubs’ web pages. We primarily contacted coaches and club chairmen, but where contact information was not available to the chairman a general club email address was used. When contact information for both chairman and club could be found, both received questionnaires.

### Knee Control+

*Knee Control+* includes a running warm-up (5 min) and six main strengthening and neuromuscular control exercises (10–15 min) to be used at every training session. In contrast to the recommendations for the original programme to be used during the warm-up, coaches were free to decide when to use the programme; for example, whether to include the exercises before training, as a warm-up, embedded in or delivered after football training. Each main exercise (squats, lunges, jump/landing technique, core strengthening, hamstring strengthening and groin strengthening) was available with 10 different variations/progressions. Within one main exercise there are both different progressions of the same exercise (such as double-leg and single-leg squats) but also different exercises (such as pelvic lifts and the Nordic hamstring exercise for hamstring strengthening). Individual exercises were organised from easy to hard. We specifically added easier as well as more advanced exercises in addition to the original programme exercises in response to coaches’ desire for a more adaptable programme that fit young as well as senior players. Additional partner and rubber band exercises were included for variation, teamwork and external resistance exercise. Coaches were advised to choose an appropriate level to begin with and to progress training over time. As examples, we provided complete programme set-ups at easy, moderate and advanced levels. With *Knee Control* we noticed barriers such as the programme taking too long or that exercises lying on the ground were not suitable in bad weather conditions. To account for this, current recommendations in *Knee Control+* are based on a set time per exercise and total training duration rather than number of repetitions, and include exercise options while standing up.

### Dissemination initiatives

In March 2021, *Knee Control+* was launched on a web page that contained films and instructions, as well as a printable programme folder in long and short formats (two pages) (https://liu.se/forskning/swipe/knakontroll-plus). The web page also included a digital lecture, covering topics such as injury risks and common injuries in football, effects of IPEPs on injury risk and recommendations on how to use *Knee Control+*.

All clubs received one printed copy each of the long-format and short-format programme folder, as well as information about the upcoming study in March 2021 by post. At the same time, all clubs and coaches received information about the programme web page and programme material via emails sent out by the research group and by the regional district FA. During the season, information about *Knee Control+* was spread via the Sport Without Injury ProgrammE (SWIPE) accounts on social media (Instagram, Facebook and Twitter, in total 6–16 posts per platform). On three occasions, coaches were invited to free digital lunch webinars, where *Knee Control+* was presented and the purpose and execution of each main exercise was discussed. During these webinars, a dialogue between researchers (HL and MH) and coaches was encouraged. Information about the webinars was spread via social media and email in advance by the research group and by the regional district FA. In total, 306 participants attended the webinars. The dissemination process is described in online supplemental figure 1.

10.1136/ip-2023-044863.supp1 Supplementary data

### Questionnaire

After the season, in October 2021, a link to a questionnaire was sent by short message service and email to 1568 coaches and club personnel, with three reminders the following 3 weeks. The questionnaire was also sent in paper format by post to all club personnel who had not yet responded after 3 weeks. The questionnaire was open for 2 months.

The questionnaire was custom made, and all five dimensions of the RE-AIM framework were covered from a club and from a coach (team) perspective. Most questions had been used in an earlier study regarding the implementation of *Knee Control* 12 and in a randomised trial.^16^ Club personnel responded to questions regarding reach, adoption, implementation and maintenance, and questions for coaches additionally covered perceived effectiveness (table 1). Club personnel also responded to three questions about the presence of policies for programme implementation, and the use and club resources for injury prevention. All survey questions can be found in tables 2–5.

**Table 1:** Dimensions of the RE-AIM framework covered in the questionnaire

Dimension of RE-AIM	Club (chairman or other administrative personnel)	Team (coach)
Reach	Whether they knew about *Knee Control+*. How they encountered the programme. (3 questions)	Whether they knew about *Knee Control+*. How they encountered the programme. (3 questions)
Perceived effectiveness		Satisfaction with *Knee Control+*. Belief in programme effects on preventing injuries and improving performance. (3 questions)
Adoption	Whether teams in the club started using *Knee Control+* during the 2021 football season. (1 question)	Whether the team started using *Knee Control+* during the 2021 football season. (1 question)
Implementation	Whether clubs had informed coaches about *Knee Control+* in the present season. (1 question)	Whether the team had done any regular training to prevent injuries in the present season. Whether the team used other measures to prevent injuries in the present season. For how long period, how often, how long time per session and how many repetitions per exercise they had used *Knee Control+,* which exercises in *Knee Control+* they used. (2+5 questions)
Maintenance	Whether clubs planned to inform coaches about *Knee Control+* in the upcoming season. (1 question)	Whether they intended to prioritise injury prevention training with *Knee Control+* in the upcoming season. (1 question)

RE-AIM, Reach, Effectiveness, Adoption, Implementation, and Maintenance.

**Table 2:** Descriptive information about club personnel and presence of policies for injury prevention

	Club personnel (n=41)
Age, mean (SD)	49.4±11.5
Sex, male/female (% male)	32/9 (78.0)
Role, chairmen/other admin (% chairmen)	28/13 (68.3)
**Policies for injury prevention (n=40)**	
*Do you have policies regarding injury prevention in the club?*	
Yes, formal policy	3/40 (7.5)
Yes, informal policy	11/40 (27.5)
Unsure	2/40 (5.0)
No	24/40 (60.0)
Is it specified in this policy which IPEP to use?*	
Yes, *Knee Control*	7/12
Yes, *Knee Control+*	0/12
Yes, *11+*	0/12
Yes, another programme	1/12
No	4/12
*What resources/possibilities do you have in the club to support injury prevention work?*†	
Possibility to consult physiotherapist/physician/naprapath	10/35 (28.6)
Help with planning and structuring injury prevention measures from, for example, physiotherapist, physician or naprapath	2/35 (5.7)
Help with execution of injury prevention initiatives from, for example, fitness coach or physiotherapist	3/35 (8.6)
Education in injury prevention measures (eg, training, recovery, nutrition, sleep)	14/35 (40.0)
Other resource/possibility	9/35 (25.7)

Values are n (%) unless otherwise stated, percentages omitted if denominator is <20.

*Two missing answers from those who had policies.

†Missing data from six club personnel.

IPEP, injury prevention exercise programme.

**Table 3:** Implementation of *Knee Control+* from the club perspective

	Club personnel
**Reach (n=35)**	
Have you previously heard about the injury prevention exercise programme Knee Control+?*	
Yes	29/35 (82.9)
No	6/35 (17.1)
*In what ways have you accessed the programme?*†‡	
Printed or digital (pdf) programme brochure	12/22 (54.5)
Lecture on Linköping University website	2/22 (9.1)
Exercise films on Linköping University website	0/22 (0.0)
Training programme on Linköping University website§	1/22 (4.5)
Digital workshop/webinar	1/22 (4.5)
Other	7/22 (31.8)
*How did you learn about Knee Control+?*†	
From contact with coaches	2/22 (9.1)
Via email or post from the research group	7/22 (31.8)
Via the regional football association website	11/22 (50.0)
Via social media (Instagram/Twitter/Facebook)	1/22 (4.5)
Other	3/22 (13.6)
**Adoption (n=22)**	
*Did teams in your club start using Knee Control+ during the 2021 season?*†	
Yes, all teams	1/22 (4.5)
Yes, some teams	8/22 (36.4)
Unsure	7/22 (31.8)
No	6/22 (27.3)
**Implementation (n=22)**	
*Did your club inform or educate coaches about Knee Control+ during the 2021 season?*†	
Yes	6/22 (27.3)
No	16/22 (72.7)
**Maintenance (n=21)**	
*Does your club intend to inform or educate coaches in Knee Control+ in the upcoming season?*¶	
Yes	12/21 (57.1)
No	9 (42.9)

Values are n (%) unless otherwise stated.

*Missing data from six club personnel.

†Missing data from seven club personnel.

‡Question where each respondent could respond more than one option, hence responses represent more than 100%.

§Digital programme films at low, moderate and advanced levels where coaches and players could exercise with support from the films.

¶Missing data from eight club personnel.

**Table 4:** Descriptive information about participating coaches

	Coaches (n=440)
Age, mean (SD)*	45.6 (7.4)
Sex, male/female (% male)	379/61 (86.1)
Experience as coach, n years mean (SD)	9.0 (6.0)
*Has your team regularly used training to prevent injuries during this season?*†	
*Knee Control*	255 (58.0)
*Knee Control+*	68 (15.5)
*11+*	9 (2.0)
Other injury prevention programme	59 (13.4)
Groin strengthening exercises	146 (33.2)
Hamstring strengthening exercises	222 (50.5)
Core strengthening exercises	329 (74.8)
One-legged knee squats	321 (73.0)
Two-legged knee squats	346 (78.6)
Lunges	359 (81.6)
Jump/landing technique	231 (52.5)
Plyometric exercises	169 (38.4)
Strength training with rubber bands	87 (19.8)
Strength training with weights	31 (7.0)
Balance training with balance board/mat	36 (8.2)
Other complementary training (yoga, fitness training…)	78 (17.7)
Other (unspecified) training	26 (5.9)
No exercises specifically to prevent injuries	27 (6.1)
*Has your team used other strategies to prevent injuries during this season?*†	
Control over player total load	122 (27.7)
Control over player restitution	70 (15.9)
Control over other wellness factors (eg, sleep, stress)	62 (14.1)
Injury registration	12 (2.7)
Screening tests of risk factors for injury	10 (2.3)
Structured return to football after injury	101 (23.0)
Taping (eg, of knee or ankle)	228 (51.8)
Use of protective equipment (eg, orthoses, shin guards)	186 (42.3)
Other measures	12 (2.7)
Did not use specific measures to prevent injuries	124 (28.2)

*Missing data from 10 people.

†Questions where each respondent could respond more than one option, hence responses represent more than 100%.

**Table 5:** Implementation of *Knee Control+* within teams

	Coaches
**Reach (n=440)**	
*Have you previously heard about the injury prevention exercise programme Knee Control+?*	
Yes	288 (65.5)
No	152 (34.5)*
*In what ways have you accessed the programme?†*	
Printed or digital (pdf) programme brochure	112/288 (38.9)
Lecture on Linköping University website	21/288 (7.3)
Exercise films on Linköping University website	55/288 (19.1)
Training programme on Linköping University website	13/288 (4.5)
Digital workshop/webinar	28/288 (9.7)
Other	116/288 (40.3)
*How did you learn about Knee Control+?†*	
Via club personnel	46/288 (16.0)
From contact with another coach	99/288 (34.4)
Via email from the research group	48/288 (16.7)
Via the regional district football organisation web page	94/288 (32.6)
Via social media (Instagram/Twitter/Facebook)	41/288 (14.2)
Took part in research study in 2020	15/288 (5.2)
Other	43/288 (14.9)
**Adoption (n=280)**	
Used *Knee Control+* during the season (parts of or the whole season)	143 (51.1)
Did not use *Knee Control*+ during the season	137 (48.9)
**Perceived effectiveness‡ (n=102)**	
Overall, I am satisfied with *Knee Control*+, 1–7 Likert scale, median (IQR)	6.0 (2.0)
I believe the injury risk of players decreased after having used *Knee Control+*	6.0 (2.0)
I believe performance of players improved after having used *Knee Control+*	5.0 (2.0)
**Implementation (n=102)**	
**Utilisation frequency (use of *Knee Control+* times/week)**	
<1 time/week	21/102 (20.6)
1 time/week	27/102 (26.5)
2 times/week	40/102 (39.2)
3 times/week	13/102 (12.7)
4 times/week or more	1/102 (1.0)
**Duration fidelity**	
Minutes spent on IPEP each session, mean±SD	13 (5)
**Utilisation fidelity**	
Duration (s) per exercise and set, mean±SD§	49 (37)
Number of sets, mean±SD¶	2.4 (1.0)
*How did you use the programme at training?*	
Same exercises throughout the season	19/102 (18.6)
Different exercises for variation	71/102 (69.6)
More advanced exercises over time	9/102 (8.8)
Individual adaptation	6/102 (5.9)
Exercises without close contact due to COVID-19	15/102 (14.7)
Used the easy programme set-up	19/102 (18.6)
Used the moderate programme set-up	13/102 (12.7)
Used the advanced programme set-up	5/102 (4.9)
**Maintenance‡ (n=102)**	
Intention to carry on prioritising *Knee Control*+ in the team in the upcoming season	7.0 (1.0)

*Respondents who had not been reached by *Knee Control+* (n=152) did not receive any more questions. Only those who had been reached by *Knee Control+* (n=288) received questions on adoption and only those who had adopted the programme (n=143) received questions about perceived effectiveness.

†Questions where each respondent could respond more than one option, hence responses represent more than 100%.

‡1–7 Likert scale from Do not agree to Totally agree.

§Five responses have been excluded due to being unreasonable (1 s and 240–999 s).

¶Twelve responses have been excluded due to being unrealistic (≥6 sets or more).

IPEP, injury prevention exercise programme.

### Statistical analyses

No sample size calculation was made, since this was a descriptive study aimed at the total population of club personnel and coaches within the regional football district.

In addition to the five dimensions of RE-AIM framework, website statistics based on Google Analytics and perceptions of what the coaches liked and disliked about the programme were covered. Results are presented descriptively with n and %. A gatekeeper principle was used, where respondents only answered relevant questions based on their previous responses; for example, only those responding that they had adopted *Knee Control+* received questions about implementation. Hence, the denominator varies between questions. Additionally, internal dropout for individual responses was present for some respondents. Free-text answers reporting how the teams had worked with injury prevention in general and injury prevention training specifically were analysed with quantitative content analysis. Here, most responses covered training modalities and training dosage, and these were categorised, and the number of responses for each category was counted. The training modalities were categorised into: ‘*Knee Control*/*Knee Control+*’, ‘strength training’, ‘core strengthening’, ‘conditioning training’, ‘coordination and balance training’, ‘dynamic mobility’, ‘jumping exercises’ and ‘stretching’. The dosage was categorised into: ‘regularly’ (including each training session, regularly and two or more times/week), ‘once/week’ and ‘sporadic’ (including irregular use and less than once/week).

### Patient and public involvement

Development of *Knee Control+* was informed by a qualitative study with coaches for female teams, where they wished for a versatile easy-to-use programme that would fit youth as well as senior players.^13^ Pilot versions of the programme were used and commented on by coaches and players in previous studies with male and female players.^16 25^ The group responsible for development of *Knee Control+* consisted of seven sports physiotherapists and one orthopaedic surgeon, whom together had experience as researchers and practitioners within sports medicine, experience from working on the field with players and as coaches at varying levels of play.

## Results

In total, 41 chairmen or other club personnel responded to the questionnaire, representing 40 different clubs. With 104 eligible clubs with contact information, the response rate was 38% from a club perspective. Among 440 responding coaches, 71 clubs out of 104 (68%) and 302 out of 442 eligible teams (68%) were represented.

### Implementation of injury prevention programmes within the clubs

Fourteen clubs (35%) reported presence of policies regarding injury prevention, the majority (11/14) of these were informal (oral) and most included the original *Knee Control* programme (table 2).

Reach of *Knee Control+* was 83% among the responding club personnel and 55% had accessed the programme via printed material from the research group (table 3). Regarding adoption, 41% of these clubs had also started using it during the 2021 season and 57% intended to educate or deliver information about *Knee Control+* to coaches in the upcoming season.

### Implementation of IPEPs among coaches

Fifty-eight percent of coaches had used *Knee Control* and 16% *Knee Control+* in the 2021 season, only 6% had not performed any training to prevent injuries, implying that 94% used some kind of preventive training (table 4). Many coaches who stated that they had used neither the complete *Knee Control* nor *Knee Control+* programme had still used several of the programme’s components, while 19% had not used any of the components in *Knee Control*/*Knee Control+*. In terms of other injury preventive measures, 58% of coaches reported use of taping, and 42% protective equipment within their team. Other resources for injury prevention are presented in online supplemental table 1.

10.1136/ip-2023-044863.supp2 Supplementary data

### RE-AIM-based evaluation of implementation of *Knee Control*+

**Figure 1:**
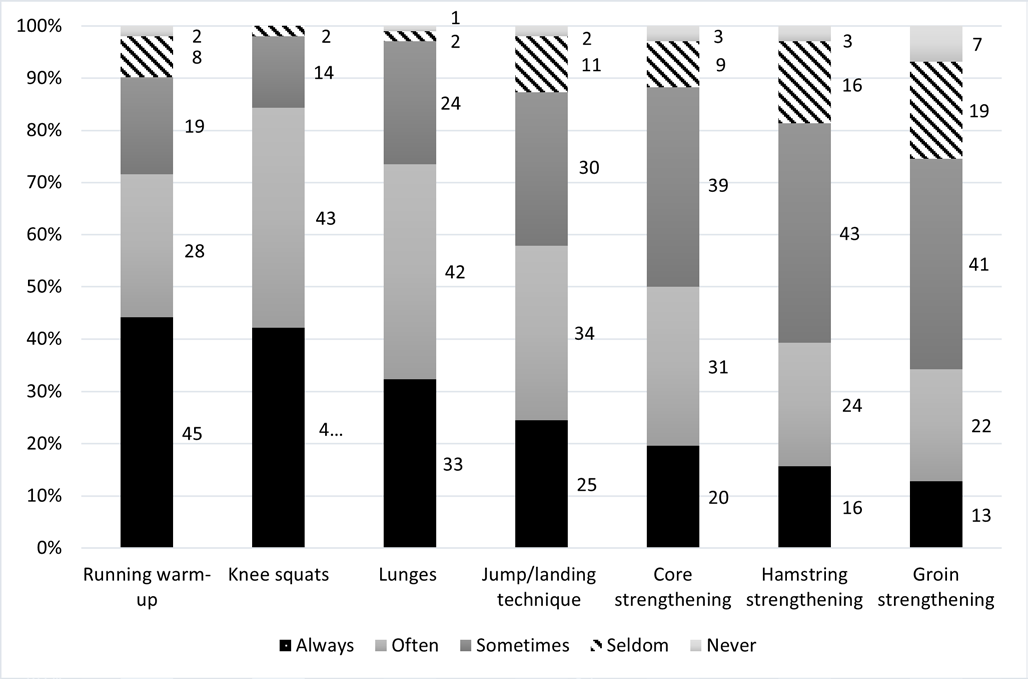
Description of how often each component of *Knee Control+* was used, as rated by coaches (n=102).

Reach of *Knee Control+* among coaches was 66% (table 5). Most had encountered the programme via another coach or via the web page of the regional district FA. Few had accessed the digital material on the university website (5%–19%). Regarding perceived effectiveness, coaches were positive and believed injury risk decreased when using *Knee Control+* (6 on a 1–7 Likert scale). Regarding adoption, 51% of those who knew about the programme had used it. Utilisation frequency varied, and 52% used the programme at least twice per week. Almost three-quarters of participants had used different exercises for variation, but only 9% had progressed with more advanced exercises over time. Use of each component of *Knee Control+* is described in figure 1.

In the optional free-text answers, 250 coaches (57%) described in detail what they did to prevent injuries. Ninety-three coaches (21%) described that they used *Knee Control*/*Knee Control+* or knee control exercises, 98 coaches (22%) used strength training and 63 coaches (14%) used conditioning training. A few other training modalities such as coordination and balance training (27 coaches, 6%), core strengthening (28 coaches, 6%), dynamic mobility (19 coaches, 4%), jumping exercises (18 coaches, 4%) and stretching (17 coaches, 4%) were mentioned. One-third (155 coaches, 35%) described their intended preventive training frequency, where 122 (28%) used it regularly, 20 (5%) used it once/week and 13 (3%) stated sporadic use.

### Experiences of the programme

On the question about what they liked about *Knee Control+*, coaches most often listed that they liked that the exercises could reduce injury risk (98 of 102, 96%) and that the players became better at performing the preventive exercises (68%). Twelve percent responded that they disliked that they had less time for football training, and 8% responded that the players did not like the exercises (online supplemental table 2).

### Website access

During the 2021 football season we had 5550 visits to the *Knee Control+* website, corresponding to 4137 new users. Most users accessed the website directly, or by using Google or Facebook. Of the 5550 visits, 1300 came from the same region that was targeted for dissemination. The coaches rated the value of the website material at a median of 6 (IQR 2.0) on a 1–7 Likert scale.

## Discussion

Reach of *Knee Control+* from club and coach perspectives was high and fair (83% and 66%, respectively), and adoption was fair (41% and 51%, respectively). Perceived effectiveness, implementation and intentions for maintenance were also high from a coach perspective. However, more active strategies may be needed in addition to the dissemination strategy used in our study to improve implementation and maintenance.

### Implementation of injury prevention training

From a club perspective, the high reach suggests that our dissemination strategy with digital and printed information about the programme, information spread via social media as well as webinars was sufficient to make them aware of *Knee Control+*. The high intention (57%) to inform or educate about *Knee Control+* for the upcoming season was also positive. Reach was fair among coaches, with 66% having heard about the programme. In an implementation evaluation of *Knee Control* in 2012,^12^ 58%–74% used *Knee Control*, which is similar to the 58% who indicated use of *Knee Control* in the present study. The fact that 94% used some kind of preventive training in 2021 is positive since it indicates that most players are reached. In contrast, a Canadian study of youth football showed use of IPEPs in only 30% of teams,^26^ and an American study in collegiate women’s football in 66% of teams.^27^

Focusing on *Knee Control+*, the 16% of coaches who had adopted and used the programme may seem low at first glance, but overall, 81% of coaches who had not used the complete *Knee Control* or *Knee Control+* had still used at least one of their components. This suggests that we need to support these coaches more in their selection of exercises since this inadequate adoption may be ineffective in preventing injuries. A previous study showed that although awareness of IPEPs was high, teams may need support to adopt and implement them.^21^ Perceived effectiveness indicated high belief in the programme’s potential to prevent injuries, which confirms earlier ratings of the *Knee Control* programme.^12^

Previous studies^12–14^ found that coaches often modify programme content or dosage, while the present study showed that the coaches that had adopted the programme often used it as recommended in terms of duration and utilisation fidelity. However, only half of teams used *Knee Control+* at least twice per week, and utilisation frequency should be improved to maximise programme effects.^6 7^ We also note that the three neuromuscular control exercises were used more often than the three strength-focused exercises. The neuromuscular control exercises were used ‘always’ by 25%–43% of coaches and the strength exercises by 13%–20% of coaches. Unfortunately, we did not collect data on the reasons for this. We evaluated intention to maintain *Knee Control+,* which was high (7 on a 1–7 Likert scale) among coaches who had started using *Knee Control+*. Long-term programme maintenance should be evaluated further. An earlier study showed benefits of having a flexible set-up with the possibility to use the exercises as warm-up, embedded or after training,^28^ and the flexible set-up may have been one reason for the positive results on training duration and utilisation fidelity as well as the high intended maintenance in the present study. The present study is unique in that it evaluated a further developed version of a well-established and efficacious programme and with specific features aiming to improve programme fidelity. The application outside the controlled context of an RCT further increases our understanding of the translation of knowledge into action, which is an understudied area.^17^

Only 3 out of 40 clubs had formal policies for IPEP use, which is concerning. Since coaches come and go, built-in structures in the club, such as policies and yearly education activities, are important to ascertain long-term IPEP use. Few clubs (6%) reported that they support coaches with resources for planning and structuring injury prevention measures that may facilitate implementation. Coaches in a previous qualitative study specifically wished for support from ‘experts’ in injury prevention.^13^ Active strategies, such as attracting media attention about the programme, offering practical workshops for coaches and personal communication with each coach, are vital for maximum effect.^21^ Only offering programme material, as part of dissemination, without implementation support may result in lower levels of implementation,^21^ and this may be one reason for the lack of policies for programme implementation and use seen in the present study. More engagement from stakeholders, such as FAs and clubs, may stress the importance of injury prevention and support the coaches with information and workshops.

### Digital strategies

Relatively few clubs (9%) and coaches (5%–19%) had visited the programme web page and accessed the digital programme material. This was surprising, considering the positive experiences of the previous *Knee Control* app.^13^ Of all visits to the web page, only 23% came from the region targeted for dissemination. Hence, we probably need to offer programme material via different media in the future with both printed and digital materials to disseminate the programme as well as offer practical workshops to support its implementation. Similarly, a study on an app to prevent ankle sprains showed challenges both with programme reach and adoption even though the app in itself received positive ratings.^29^ It was emphasised that it is important to know the target population better to succeed with prevention and for it to be worthwhile to develop more apps.^29^ To succeed with future efforts to implement *Knee Control+*, we may therefore need to engage the target population even more and adapt programme material and media to better accommodate coaches’ needs.

### Strengths and limitations

One strength of the present study was that we used all five dimensions of the RE-AIM framework to evaluate *Knee Control+* implementation in a large sample of clubs and coaches. Another strength was that the evaluation occurred in an applied setting, within a regional football district and not as part of an RCT, which increases the external validity of our findings. We believe the results are generalisable to similar contexts, primarily amateur football in Sweden, but also neighbouring countries with similar contexts where coaches primarily work on a voluntary basis.

Some limitations should be mentioned. First, only 38% of clubs and 68% of teams within the regional district were represented and clubs were only represented by 41 club personnel. We reminded all non-responding club personnel and coaches on three occasions and believe the response rates are acceptable when compared with similar studies with response rates between 24% and 36%.^12 26 27^ However, there is a risk that only the most motivated coaches with knowledge about *Knee Control* or *Knee Control+* participated, potentially overestimating the programme implementation. Since the study was only carried out in one football district, the results are not generalisable to other football districts unless similar dissemination would take place within these. Second, the study was cross-sectional and there is a risk for recall bias regarding programme use across the season. Third, some club personnel and coaches may have found it difficult to distinguish between *Knee Control* and *Knee Control+*. Hence, programme reach, perceived effectiveness, adoption, implementation and maintenance may be slightly underestimated or overestimated. However, from our perspective, regular use of any evidence-based programme (eg, *Knee Control, Knee Control+* or *11+*) is positive. Since *Knee Control+* builds on the original programme, we see no reason to change from *Knee Control* to *Knee Control+* for teams who find the original programme feasible. Fourth, the questionnaire has not undergone formal validation. We have used similar questions in previous studies^12 16^ and have tested them with coaches, and we believe they have high face validity. Fifth, the study relied on self-reported data, which have an inherent risk of social desirability bias with clubs and coaches potentially responding with the answers they believe are expected. Sixth, this study relied on the dissemination of the programme to clubs and coaches primarily through us as researchers and we did not intervene to improve implementation through other stakeholders such as the FA, parents or media. Even though we have evaluated the implementation of the programme per se, there is more to learn about the implementation infrastructure and the programme users to be able to further improve implementation.

## Conclusion

The reach of *Knee Control+* was high and adoption was fair in amateur football clubs. There was a lack of policies for preventive programme use and few resources for supporting coaches’ implementation. Dissemination of programme material should probably be accompanied by active strategies for implementation of injury prevention training within the clubs. From a coach perspective, reach, perceived effectiveness, adoption, implementation and planned maintenance were positive, but further studies are needed to follow-up long-term maintenance of *Knee Control+*.

## Data Availability

All data relevant to the study are included in the article.
