# Supplementary material for: Implementation of the injury prevention exercise programme Knee Control+: a cross-sectional study after dissemination efforts within a football district
Source: Inj Prev. 2023 May 31;29(5):399–406. doi: 10.1136/ip-2023-044863 (PMC10579513; doi:10.1136/ip-2023-044863)
Supplement: Supplementary data [file ip-2023-044863supp001.pdf]

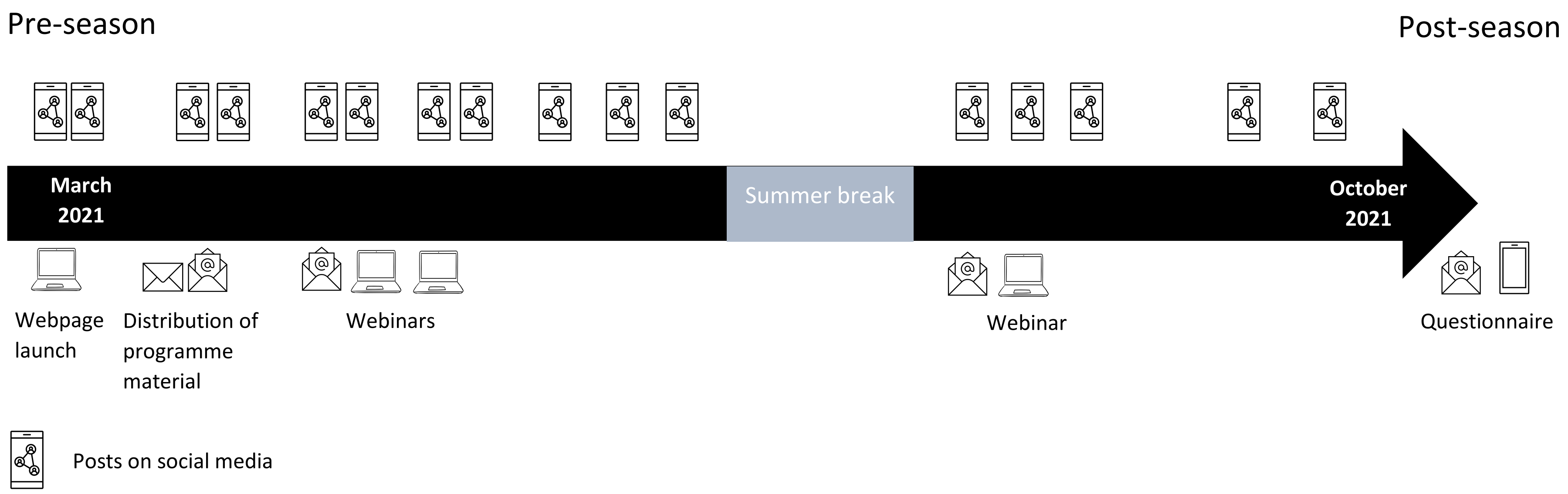

Supplementary figure 1. Illustration of programme dissemination efforts during the 2021 football season
