# Supplementary material for: Implementation of the injury prevention exercise programme Knee Control+: a cross-sectional study after dissemination efforts within a football district
Source: Inj Prev. 2023 May 31;29(5):399–406. doi: 10.1136/ip-2023-044863 (PMC10579513; doi:10.1136/ip-2023-044863)
Supplement: Supplementary data [file ip-2023-044863supp002.pdf]

Supplementary tables

Supplementary table 1. Coach resources/possibilities for help with injury prevention

What resources/possibilities do you have in the club to support injury prevention work?	Coaches (n =440)
Possibility to consult physiotherapist/physician/naprapath	90 (20.5)
Help with planning and structuring injury prevention measures from e.g., physiotherapist, physician, naprapath	48 (10.9)
Help with execution of injury prevention initiatives from e.g., fitness coach or physiotherapist	82 (18.6)
The club offers education in injury prevention training	134 (30.5)
The club offers education in other injury prevention measures (e.g., recovery, nutrition, sleep)	60 (13.6)
Other resource/possibility (free text response)	115 (26.1)
No resource	77 (17.5)

Each respondent could respond more than one option; hence responses represent more than 100%

Supplementary table 2. Description of what coaches liked and disliked about *Knee Control+*

	Coaches (n =102)
What did you like about Knee Control+?	
The exercises could reduce player injury risk	98 (96.1)
The players became better at performing the preventive exercises	69 (67.6)
The exercises could be varied over time	55 (53.9)
The ball was used during some exercises	47 (46.1)
Structured warm-up	42 (41.2)
Some exercises could be done with a partner	37 (36.3)
Exercises were a break from normal training	36 (35.3)
The players liked the exercises	27 (26.5)
We used equipment during some exercises	21 (20.6)
The players improved as football players from doing preventive exercises	19 (18.6)
The players could compete in some exercises	13 (12.7)
The parents liked the exercises	4 (3.9)
Nothing, I did not like the programme/exercises	0 (0.0)
What did you not like about Knee Control+?	0 (0.0)
We had less time for football training	12 (11.8)
The players did not like the exercises	8 (7.8)
The exercises were boring	3 (2.9)
The programme was too long	6 (5.9)
The players experienced pain during preventive training	4 (3.9)
The exercises were too hard/difficult	2 (2.0)
The programme was too short	2 (2.0)
I did not understand why the players should do preventive training	1 (1.0)
The exercises were too easy	1 (1.0)
The exercises did not have anything to do with football	1 (1.0)
The parents did not like the exercises	1 (1.0)
Nothing, I liked the programme/exercises	69 (67.6)
